# Modulation Index Adjustment for Recovery of Pure Wavelength Modulation Spectroscopy Second Harmonic Signal Waveforms

**DOI:** 10.3390/s17010163

**Published:** 2017-01-15

**Authors:** Wei Wei, Jun Chang, Qiang Wang, Zengguang Qin

**Affiliations:** School of Information Science and Engineering and Shandong Provincial Key Laboratory of Laser Technology and Application, Shandong University, Jinan 250100, China; zero931103056@126.com (W.W.); iamwq1989@gmail.com (Q.W.); qinzengguang@sdu.edu.cn (Z.Q.)

**Keywords:** water, fiber optics sensors, diode lasers, wavelength modulation spectroscopy, modulation index adjustment

## Abstract

A new technique of modulation index adjustment for pure wavelength modulation spectroscopy second harmonic signal waveforms recovery is presented. As the modulation index is a key parameter in determining the exact form of the signals generated by the technique of wavelength modulation spectroscopy, the method of modulation index adjustment is applied to recover the second harmonic signal with wavelength modulation spectroscopy. By comparing the measured profile with the theoretical profile by calculation, the relationship between the modulation index and average quantities of the scanning wavelength can be obtained. Furthermore, when the relationship is applied in the experimental setup by point-by-point modulation index modification for gas detection, the results show good agreement with the theoretical profile and signal waveform distortion (such as the amplitude modulation effect caused by diode laser) can be suppressed. Besides, the method of modulation index adjustment can be used in many other aspects which involve profile improvement. In practical applications, when the amplitude modulation effect can be neglected and the stability of the detection system is limited by the sampling rate of analog-to-digital, modulation index adjustment can be used to improve detection into softer inflection points and solve the insufficient sampling problem. As a result, measurement stability is improved by 40%.

## 1. Introduction

Tunable diode laser spectroscopy (TDLS) has become the primary technology for most kinds of gas detection, particularly for trace gas concentration detection [1,2,3,4,5]. Earlier, it was mainly the fundamental rotation-vibration absorption lines that applied TDLS to deal with the mid-infrared (MIR) [6,7,8,9,10,11,12]. However, the need of frequently cooling the lasers and detectors restricted the widespread use of such systems. The application of near-infrared (NIR) components, which were originally developed for the optical fiber telecommunications area, provided distributed feedback (DFB) lasers and indium gallium arsenide (InGaAs) photodiodes. Furthermore, the application of these components effectively solved the problem of weaker absorption lines of the target gases [13,14,15,16]. The distributed feedback diode lasers (DFB-DLs) used in TDLS have the benefits of being compact, stable and allowing temperature control. Compared with other conventional gas sensing methods, in situ measurement provides real-time data transmission and non-touch measurement. As lasers have become cheaper and more available in practical application, TDLS in the NIR region has become more attractive.

TDLS with wavelength modulation spectroscopy (WMS) enables alternating-current (AC) detection at some frequency of choice and the use of a lock-in amplifier (LIA) for better signal recovery [17,18,19,20]. Each harmonic signal is proportional to the concentration of target gas and can be measured with the LIA. An important advantage of this technique is that detection is shifted to higher frequencies. As a result, the laser excess noise (1/f noise) becomes negligible. Furthermore, the use of the LIA can further reduce the noise outside the bandwidth. As a result, WMS is from one to two orders of magnitude more sensitive than direct detection. The WMS method also provides advantages in suppressing interfering background signals and providing more sensitive signal recovery when detected light is weak.

In conventional TDLS with wavelength modulation (WM), the DFB-DL is modulated by a combination of a low frequency ramp (0.1–1000 Hz commonly) [6,21,22] and a high frequency sinusoidal (>1 kHz commonly). Other than wavelength modulation for tunable diode lasers, the drive current signal produces amplitude modulation (AM) at the same time. This interaction between modulation of the transmitted laser power and absorption of target gas produces residual amplitude modulation (RAM) signals at the fundamental sinusoid frequency and its higher harmonics. In WMS detection, the detected signals mainly derive from the WM of the laser, whereas the effect caused by amplitude modulation (AM) is an unwanted effect and is adjusted to recover absorption line shape to extract information [19,23,24].

Application of first harmonic detection in TDLS with WM techniques is mainly limited by the presence of a large unwanted background signal, caused by the AM of the laser. This high background signal limits amplification of signal-processing modules and deteriorates sensitivity of the detection system. Second harmonic detection is widely used to solve this problem, resulting in improved signal resolution and sensitivity [6,19,25]. However, second harmonic detection has problems, including signal waveform distortion caused by AM and a small background signal arising from the RAM [24].

The current technique used for RAM effect elimination and waveforms recovery mainly applies double-beam-double-detector schemes. A π phase difference is ensured between two paths and amplitudes of the two paths are balanced. Finally, the two paths are combined and the RAM effect is suppressed [6,26,27]. In addition, the electrical nulling method [19,28,29,30] and a three-sections quantum cascade laser (QCL) [25] are also mentioned for RAM suppression. However, the two-beams structure has been proven to be unstable, with poor accuracy, poor reproducibility and high cost in our lab and in other papers as well.

In the work reported here, we came up with a new method for pure WMS signal recovery, which overcomes the defects mentioned before, is unlimited by modulation value (unlimited by profile distortion) [6] and is more attractive. A key parameter in determining the exact form of the recorded signals is the modulation index *m* (defined by the ratio of the wavelength modulation depth Δν and the half width of the transition lineshape γ), i.e., *m* = Δν/γ. The modulation index *m* is set as constant during the experiment when the standard procedure for traditional WMS is applied. However, the modulation index *m* is adjusted during the experiment in the proposed method. By adjusting the modulation index *m* point-by-point during the slow scan of the laser center frequency, detection signal waveforms can be recovered. This paper is illustrated mainly with the second harmonic signal. The experimental system and theoretical calculation are introduced. Validity through measurements on water compared to theoretical traces derived from HITRAN data is demonstrated as well.

Besides, the modulation index adjustment method can be used in other aspects which involve profile improvement and another example is presented as well.

## 2. Analytical Treatment for Waveforms Recovery

Firstly, the principles of gas detection in traditional TDLS with WMS will be presented. Once the radiation of the diode laser overlaps with a rotation/vibration transition of gas, absorption will generate and this results in light intensity attenuation. The transmitted intensity *I*_out_(t) at simultaneous time *t* associated with gas transition in a gas cell is given by the Beer-Lambert law [4,5,6]:

(1) $$I_{\mathrm{out}}(t)=I_{\mathrm{in}}\cdot \exp\{-\alpha [\nu (t)]CL\}\approx I_{in}\cdot \{1-\alpha [\nu (t)]CL\}$$

where *I*_in_ is incident intensity, α[ν(*t*)] (cm^−1^) is the absorption coefficient at instantaneous frequency ν(*t*), *L* (cm) is the optical path length, and *C* is the concentration of target gas (ppm; i.e., target gas density ratio in the mixed gas) in the gas cell. The approximation of Equation (1) is valid for small absorbance, i.e., α[ν(*t*)]CL << 1.

For the gas absorption line under atmospheric pressure (0.1 MPa) and normal temperature (298 K), Lorentzian profile is applicable, where the absorption coefficient α[ν(*t*)] is defined by:

(2) $$\alpha [\nu (t)]=\frac{\alpha_{0}}{1+{\left[\frac{\nu (t)-\nu_{0}}{\gamma }\right]}^{2}}$$

where α_0_ and ν_0_ are the absorption coefficient and optical frequency at the absorption line center, respectively. γ is the half-width of the transition lineshape. Consider the laser modulation obtained by combining a small sinusoidal AC signal of angular frequency ω and a constant direct-current (DC) bias to drive a diode laser maintained at constant temperature. Other than WM for tunable diode lasers, the drive current signal produces AM at the same time. Then simultaneous frequency ν(*t*) and incident intensity *I*_in_(*t*) can be written as:

(3) $$\nu (t)=\nu_{\mathrm{ave}}+\Delta \nu \cdot \cos\omega t$$

(4) $$I_{\mathrm{in}}(t)=I_{\mathrm{ave}}+\Delta I\cdot \cos(\omega t+\varphi )+\Omega I\cdot \cos(2\omega t+\phi )$$

where average quantities ν_ave_ and *I*_ave_ vary slowly as the laser scans. Δν is the amplitude of WM. Δ*I* is the amplitude of AM, which is determined by the slope efficiency of the laser (Power vs. current), and Ω*I* is the nonlinear AM amplitude [24]. φ is the WM/AM phase separation, and ϕ is the phase separation of the nonlinear WM/AM. This allows Equation (1) to be rewritten as:

(5) $$I_{\mathrm{out}}(t)=[I_{\mathrm{ave}}+\Delta I\cdot \cos(\omega t+\varphi )+\Omega I\cdot \cos(2\omega t+\phi )]\cdot \{1-\alpha [\nu (t)]CL\}$$

Full expression of laser intensity *I*_out_(*t*) is obtained by developing the function in Fourier cosine series [22]:

(6) $$I_{\mathrm{out}}[\nu (t)]=[I_{\mathrm{ave}}+\Delta I\cdot \cos(\omega t+\varphi )+\Omega I\cdot \cos(2\omega t+\phi )]\cdot [1+\sum_{k=0}^{+\infty }H_{k}(\nu_{\mathrm{ave}},\Delta \nu )\cos(k\omega t)]$$

where functions *H_k_*(ν_ave_, Δν) are given by [24]:

(7) $$H_{0}(\nu_{\mathrm{ave}})=\frac{1}{2\pi }\int_{-\pi }^{+\pi }\left[-\alpha (\nu_{\mathrm{ave}}+\Delta \nu \cdot \cos\omega t)\cdot CL\right]dt\;k=0$$

(8) $$H_{k}(\nu_{\mathrm{ave}})=\frac{1}{\pi }\int_{-\pi }^{+\pi }[-\alpha (\nu_{\mathrm{ave}}+\Delta \nu \cdot \cos\omega t)\cdot CL]\cdot \cos(k\omega t)dt\;k>0$$

Then, the expression of transmitted intensity can be written as:

(9) $$I_{\mathrm{out}}[\nu (t)]=\sum_{k=0}^{+\infty }S_{k}(\nu_{\mathrm{ave}})\cos(k\omega t)$$

where *S_k_*(ν_ave_) is the amplitude of the *k*th harmonic signal. Then, the expression of the first harmonic signal (i.e., 1f) is given by:

(10) $$\begin{array}{lll} S_{1}(\nu_{\mathrm{ave}}) & = & I_{\mathrm{ave}}\cdot H_{1}(\nu_{\mathrm{ave}})+\frac{\Delta I\cdot \cos\varphi }{2}[2+2H_{0}(\nu_{\mathrm{ave}})+H_{2}(\nu_{\mathrm{ave}})]\\ & + & \frac{\Omega I\cdot \cos\phi }{2}[H_{1}(\nu_{\mathrm{ave}})+H_{3}(\nu_{\mathrm{ave}})] \end{array}$$

Expression of the second harmonic signal (i.e., 2f) is given by:

(11) $$\begin{array}{lll} S_{2}(\nu_{\mathrm{ave}}) & = & I_{\mathrm{ave}}\cdot H_{2}(\nu_{\mathrm{ave}})+\frac{\Delta I\cdot \cos\varphi }{2}[H_{1}(\nu_{\mathrm{ave}})+H_{3}(\nu_{\mathrm{ave}})]\\ & + & \frac{\Omega I\cdot \cos\phi }{2}[2+2H_{0}(\nu_{\mathrm{ave}})+H_{4}(\nu_{\mathrm{ave}})] \end{array}$$

From Equation (10), we can see that the large unwanted background signal is generated from Δ*I*∙cosφ∙*H*_0_, and distortion of the signal is caused by Δ*I*∙cosφ∙*H*_2_ for the first harmonic signal. From Equation (11), we can see that the second Fourier component *H*_2_ contributes mostly to *S*_2_, and AM is responsible for the distortion of the signal compared with the case of pure wavelength modulation, as shown by the presence of Δ*I*. At the same time, a small background signal caused by Ω*I* (i.e., RAM effect) distorts the signal as well.

Then, the principles of our signal recovery method will be discussed. As mentioned before, a key parameter in determining the form of WMS signals is the modulation index *m*. So change of the modulation index is used to recover pure WMS waveforms. While gas detection is usually operated in a relatively stable environment, the change of temperature and pressure is limited and half width of the transition lineshape γ can be seen as a constant value. So adjustment of the modulation index would be realized by adjustment of the wavelength modulation depth Δν, or adjustment of the amplitude modulation Δ*I*. For convenience, *R*_1_(ν_ave_) is introduced to define the relationship between the wavelength modulation and the amplitude modulation, i.e., Δ*I* = *R*_1_(ν_ave_)∙Δν. Furthermore, *R*_2_(ν_ave_) is introduced to define the relationship between the wavelength modulation and the nonlinear amplitude modulation, i.e., Ω*I* = *R*_2_(ν_ave_)∙Δν.

When the modulation index is adjustable and the wavelength modulation depth Δν becomes another variable, Equation (11) can be rewritten as:

(12) $$\begin{array}{lll} S_{2}(\nu_{\mathrm{ave}},\Delta \nu ) & = & I_{\mathrm{ave}}\cdot H_{2}(\nu_{\mathrm{ave}},\Delta \nu )+\frac{R_{1}(\nu_{\mathrm{ave}})\cdot \Delta \nu \cdot \cos\varphi }{2}[H_{1}(\nu_{\mathrm{ave}},\Delta \nu )+H_{3}(\nu_{\mathrm{ave}},\Delta \nu )]\\ & + & \frac{R_{2}(\nu_{\mathrm{ave}})\cdot \Delta \nu \cdot \cos\phi }{2}[2+2H_{0}(\nu_{\mathrm{ave}},\Delta \nu )+H_{4}(\nu_{\mathrm{ave}},\Delta \nu )] \end{array}$$

**Figure 1:**
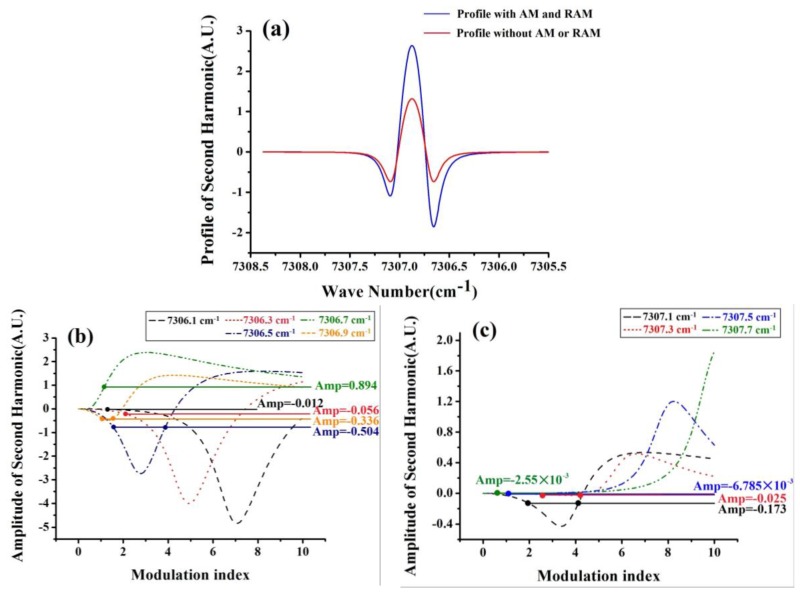
Amplitude of the second harmonic with amplitude modulation (AM) and residual amplitude modulation (RAM) is greater than that without AM or RAM in (**a**), so we can learn that the profile with AM and RAM can be adjusted to the profile without AM or RAM. Relationships between the amplitude of the second harmonic and the modulation index *m* are given in (**b**,**c**) by solid lines, so we can learn that not only can the profile be adjusted, the value of the modulation index for the adjustment can be obtained.

From the equations, we can see that the consequence of WMS not only depends on ν_ave_, it depends on Δν as well. Figure 1a shows the profile of the second harmonic with and without AM or RAM respectively(where Δ*I* and Ω*I* in Equation (5) are removed when the profile without AM or RAM is presented, i.e., red line, or Equation (12) is given by: *S*_2_(ν_ave_,Δν) = *I*_ave_∙*H*_2_(ν_ave_,Δν), at a temperature of 296 K, pressure of 1 atm, absorption line center of 1368.597 nm, water vapor concentration of 500 ppm, effective optical length of 3 m and modulation index of *m* = 2.2. We can see that with AM and RAM, the amplitude of the second harmonic is greater than the amplitude without AM or RAM throughout the spectral range. As there will be almost no second harmonic signal when the modulation index is zero, it is able to adjust the profile with AM to the profile without AM. Figure 1b,c shows relationships between the amplitude of the second harmonic and the modulation index *m* at some specific wavelength with AM and RAM. The amplitude of the second harmonic without AM or RAM is given as well, and the modulation index is set as *m* = 2.2 this time. The intersections of solid lines (with AM and RAM) and dotted lines (without AM or RAM) give the value of the modulation index for signal waveform recovery. So when a given modulation index *m* at specific scanning average quantities ν_ave_ is applied, the amplitude of the second harmonic with AM and RAM can be adjusted to the amplitude of the second harmonic without AM or RAM. Although AM and RAM, which are generated by characteristics of the diode laser, cannot be removed, the effect caused by AM and RAM can be suppressed when the pure WMS signal waveforms are recovered. As a result, WMS waveforms can be used for target gas analysis more conveniently.

## 3. Presentation of Experimental Setup

**Figure 2:**
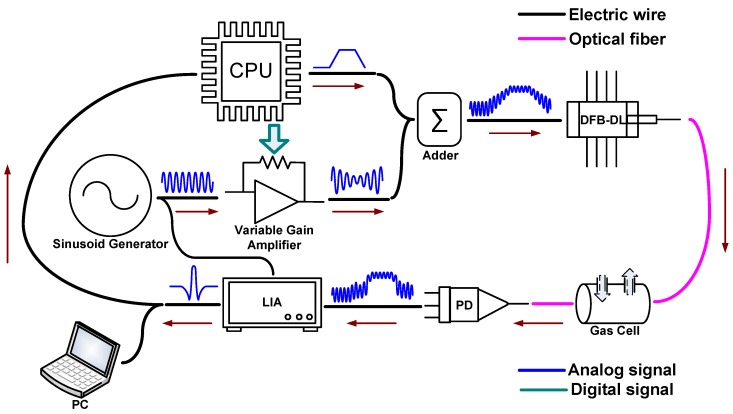
Experimental setup.

Our system for the trace measurement of water vapor used a distributed feedback diode laser (DFB-DL; DFB-1368-F-N; Wuhan 69 Sensor Technology, Wuhan, China) with an emission wavelength of 1368 nm as the light source. The experimental setup is shown in Figure 2. A 0.3 Hz trapezoidal scanning signal is generated by CPU (LTC1758; NXP, Eindhoven, the Netherlands), which determines average quantities of the scanning wavelength ν_ave_. A faster ramp could reduce the signal processing time and increase average times in a certain period of time. However, bandwidth of the LIA will be deteriorated, resulting in a lower signal-to-noise ratio (SNR), while slower ramp means higher SNR and less averaging times. A 8.1 kHz sinusoidal modulation signal is generated by a sinusoid generator (FY2300, Feiyi, Zhengzhou, China). As described before, the modulation index should be adjusted according to average quantities of wavelength. So the modulation signal passes through a variable gain amplifier for modulation index adjustment, and the magnification of the amplifier is controlled by the CPU continuously. Then, the two signals are mixed at the adder as the drive signal for DFB-DL. The output of the DFB-DL is coupled into optical fiber and goes to the gas cell (path length 20 cm between collimators). Longer path length of the gas cell commonly means better SNR and higher resolution. So some efficient structures such as the Herriot cell, are recommended for performance improvement. Output of the gas cell is detected by an InGaAs photo-detector (PD; BF14-PD300-F-N; Wuhan 69 Sensor Technology, Wuhan, China). The PD converts light into an electrical signal and sends it to a lock-in amplifier (LIA; Model 7230 DSP Lock-in Amplifier; AMETEK, Berwyn, PA, USA). With the help of a demodulation signal given by a sinusoid generator, the LIA demodulates the input signal and outputs the amplitude of the signal with the given frequency. Finally, the demodulated signal is sent back to CPU or to a PC for acquisition and data processing.

## 4. Measurement Results

**Figure 3:**
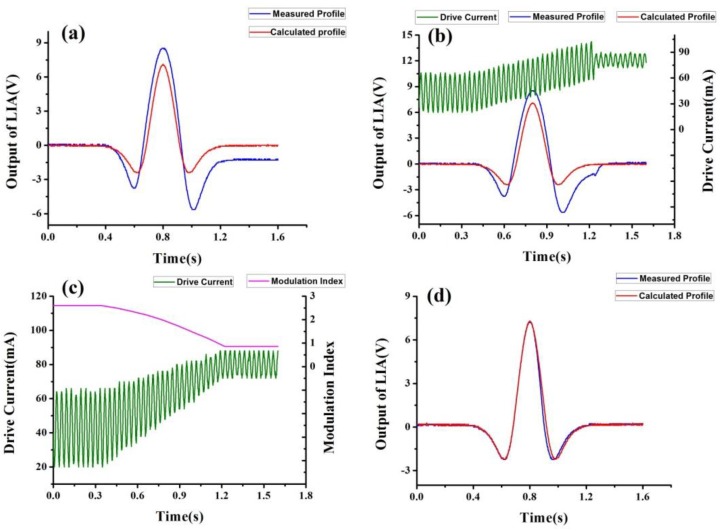
(**a**) shows the oscilloscope traces of the output of the lock-in amplifier (LIA) with amplitude modulation (blue line) and the signal sent out by digital-to-analog (DA) of our microcontroller (red line) respectively; (**b**) shows the oscilloscope traces of the output of the LIA (blue line), the signal sent out by DA (red line) and the drive current (green line); (**c**) shows the oscilloscope trace of the drive current (green line) and the rule of the modulation index over time (pink line); (**d**) shows the oscilloscope traces of the output of the LIA with modulation index adjustment (blue line) and the signal sent out by DA (red line) respectively.

In actual measurement, modulation index calculation in advance would be difficult and not fully effective due to the parameters’ diversity of different DFB-DLs. So we came up with a method of modulation index adjustment for signal waveforms recovery in actual measurement, which applies the same principle and is more effective. Firstly, we should get the profile we need, which here is the ideal 2f signal by calculation. Then the profile will be sent out by hardware, such as a digital-to-analog (DA) microcontroller or a data-acquisition card (DAQ) with Labview. As the ideal 2f signal is sent by DA or DAQ, it can be directly detected as a voltage signal by hardware, such as an oscilloscope. Secondly, by comparing the measured signal demodulated by the LIA and the desired signal sent by hardware at the same time as shown in Figure 3a, such as by the two channels of an oscilloscope, we can adjust the modulation index at several points (at different ν_ave_) accordingly as shown in Figure 3b. Accuracy of the modulation index in real time determines the proximity to the profile of the ideal signal. Furthermore, the more points (different ν_ave_) we use for modulation index adjustment, the profile of the measured signal becomes more similar to the profile of the ideal signal. To obtain better observation and understanding, the third channel of the oscilloscope-drive current is shown as well. The difference between Figure 3a and Figure 1a is caused by tuning range limitation of DFB-DL. Thirdly, we can obtain the relationship between the modulation index and ν_ave_, and when this relationship is applied as shown in Figure 3c, the signal of WMS will be normal again as shown in Figure 3d. Although AM and RAM, which are generated by characteristics of the diode laser, cannot be removed, the effect caused by AM and RAM can be suppressed by pure WMS signal waveforms recovery.

By comparing Figure 3a,d, we can find out that the close agreement between the measured and theoretical profiles validates the use of the modulation index adjustment method to recover the second harmonic signal waveform.

**Figure 4:**
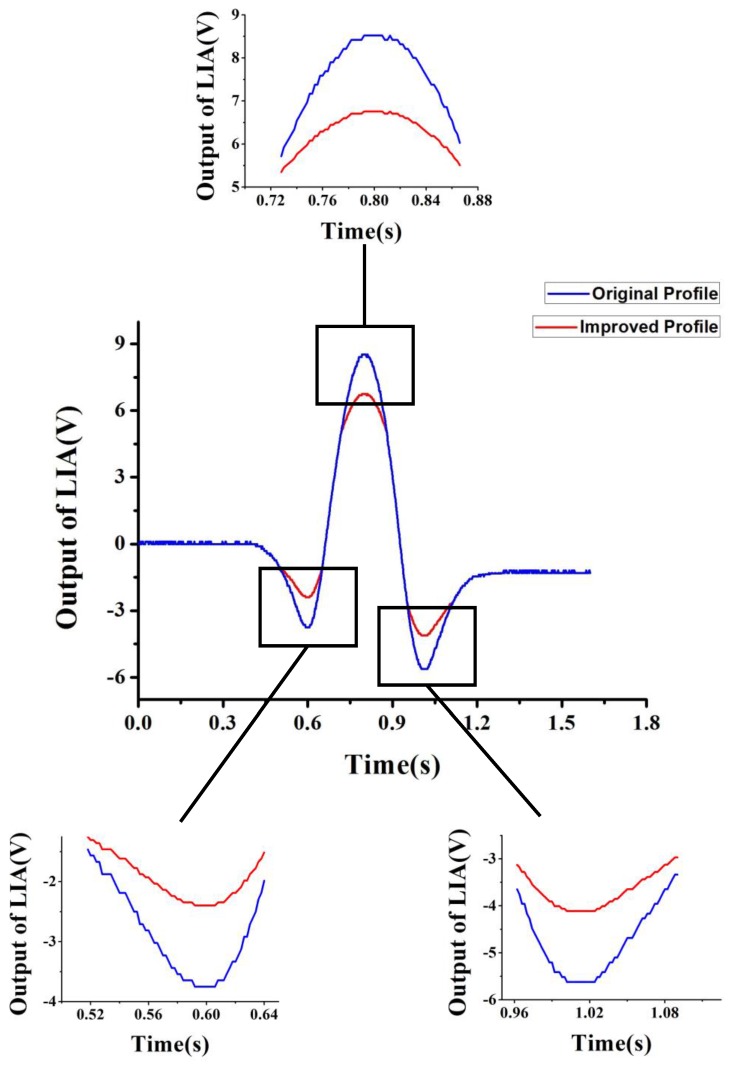
Original profile of the second harmonic (blue line) and the improved profile with modulation index adjustment (red line). Softer inflection points are shown in detail.

Besides, the method of modulation index adjustment can be used in many aspects which involve waveform adjustment. For instance, in practical applications with WMS detection, signal recovery is sometimes not necessary, the RAM effect can be neglected and the stability of the detection system is frequently limited by the sampling rate of analog-to-digital (AD). It is mostly caused by insufficient sampling at the peak of the profile, especially when the scanning ramp frequency is relatively high. With the four steps mentioned above, we can send out a waveform with softer inflection points. By adjusting the modulation index, inflection points of the profile are improved and more sampling points can land on them as shown in Figure 4. As a result, the stability of the detection system will be increased. In our experiment, when measurement concentration ranges from 5 ppm to 500 ppm, temperature is 296 ± 0.5 K, humidity is 45% ± 0.5%, pressure is 1 atm, modulation sinusoidal frequency is 8.1 kHz, sweep ramp frequency is 0.3 Hz, effective optical length is 3 m with a Herriot gas cell, resolution of the system is 0.06 ppm, and measurement stability is improved from 0.3 ppm to 0.18 ppm with waveform improvement applied. Although the amplitude of the improved profile gets smaller than that of the original profile, which results in the detection sensitivity being reduced by 20%, the measurement stability is improved by 40% because more sampling points can land on the peaks of the profile.

## 5. Conclusions

A new method of modulation index adjustment is studied in TDLS with WMS to recover pure harmonic signal waveforms and eliminate the effect brought by AM and RAM. As laser intensity also changes with the driving current, AM (or intensity modulation) and RAM are generated by the tunable diode laser for WMS detection. As a result, signals would be distorted and absorption lineshape recovery becomes more difficult. As the modulation index is a key parameter in determining the exact form of the signals generated by the technique of WMS, the method of modulation index adjustment is applied to recover the signal. By comparing the measured profile with the theoretical profile by calculation, the relationship between the modulation index and average quantities of the scanning wavelength can be obtained. Furthermore, when the relationship is applied for gas detection, the results show good agreement with the theoretical profile. At the same time, the experimental setup with the new method applied is shown and illustrated. As there is no additional light path, the new structure for WMS signal waveforms recovery is more stable and with better performance than the traditional double-beams structure.

Besides, the new method of modulation index adjustment can be used in many other aspects which also involve profile improvement and an example is shown in the end. As the stability of the detection system is generally limited by the sampling rate of AD, modulation index adjustment can be used to improve detection into softer inflection points. With the same method used in RAM effect suppression, the profile is improved and the insufficient sampling problem can be solved. As a result, measurement stability is improved from 0.3 ppm to 0.18 ppm by the experiment.

The AM or RAM effect can be suppressed when characteristics of the diode laser (such as the amplitude of AM Δ*I* and the nonlinear AM amplitude Ω*I*) are optimized. However, the side effect of the tunable diode laser cannot be removed. So the AM and RAM effect cannot be eliminated completely as long as the diode laser is applied for optical gas detection. In addition, external amplitude modulation by an electro-optic modulator has been used by Zhu [31] for AM and RAM elimination. However, the disadvantages include linear modulation range limitation, large volume and high cost, and especially phase fluctuation between the injection current and the demodulation signal brought about by the electro-optic modulator limit in its application in TDLAS. The method proposed by us can serve as a more effective and realizable way.
